# Awareness of metabolic dysfunction–associated steatotic liver disease (MASLD) in 4 major cities in the United States

**DOI:** 10.1097/HC9.0000000000000704

**Published:** 2025-05-06

**Authors:** Jeffrey V. Lazarus, Trenton M. White, Alina M. Allen, Silvana Pannain, Naim Alkhouri, Meena B. Bansal, Michael Charlton, Brett E. Fortune, Yehuda Handelsman, Scott Isaacs, Ira M. Jacobson, Sonal Kumar, Melina I. Manolas, Mazen Noureddin, Mary E. Rinella, Norah Terrault, Ayman El-Mohandes

**Affiliations:** 1 City University of New York Graduate School of Public Health & Health Policy (CUNY SPH), New York City, New York, USA; 2 Barcelona Institute for Global Health (ISGlobal), Barcelona, Spain; 3 Department of Medicine, Division of Gastroenterology and Hepatology, Mayo Clinic, Rochester, Minnesota, USA; 4 Department of Medicine, Section Endocrinology, Diabetes and Metabolism, University of Chicago, Chicago, Illinois, USA; 5 Fatty Liver Program, Arizona Liver Health, Phoenix, Arizona, USA; 6 Division of Liver Diseases, Icahn School of Medicine at Mount Sinai, New York City, New York, USA; 7 Department of Medicine, Center for Liver Diseases, University of Chicago, Chicago, Illinois, USA; 8 Department of Medicine, Division of Hepatology, Montefiore Einstein, New York City, New York, USA; 9 Metabolic Institute of America, Los Angeles, California, USA; 10 School of Medicine, ﻿Emory University, Atlanta, Georgia, USA; 11 ﻿Department ﻿of Hepatology, NYU Langone Health, New York City, New York, USA; 12 Division of Gastroenterology and Hepatology, Weill Cornell Medical College, New York City, New York, USA; 13 Department of Medicine, Division of Endocrinology, Montefiore Einstein, New York City, New York, USA; 14 Houston Research Institute, Houston, Texas, USA; 15 Division of Gastroenterology, Hepatology and Nutrition, Pritzker School of Medicine, University of Chicago, Chicago, IL; 16 Keck School of Medicine, University of Southern California (USC), Los Angeles, California, USA

**Keywords:** diabetes, MASH, MASLD, noncommunicable diseases, prevention, risk factors, urban health policy

## Abstract

**Figure:**
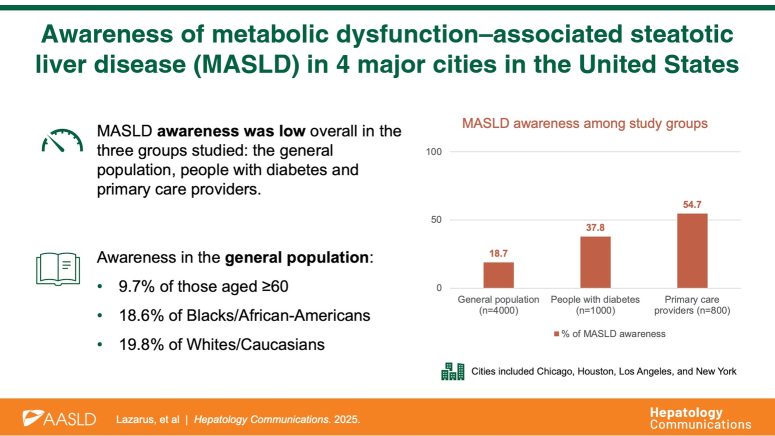

## INTRODUCTION

Metabolic dysfunction–associated steatotic liver disease (MASLD), known until mid-2023 as NAFLD,^1^ and its more severe form, metabolic dysfunction–associated steatohepatitis (MASH), are characterized by liver fat accumulation, often comorbid with obesity, type 2 diabetes, hypertension, and dyslipidemia—conditions associated with insulin resistance and related metabolic risk factors.^2^ MASLD, part of the spectrum of fatty liver diseases (now collectively called steatotic liver disease), is the most common chronic liver disease globally, affecting ~30% of adults and over 10% of children—a 50% increase in prevalence over 3 decades.^3^

Despite this high and increasing prevalence, public awareness remains low, which led to awareness being selected as 1 of 6 priority areas in the global MASLD/MASH research agenda.^4^ In 2016, an estimated 6.3% of individuals with MASLD in the United States were aware of their condition, even lower among younger adults and non-Hispanic Black populations.^5^ Many remain unaware as MASLD is often asymptomatic until progressing to advanced stages (eg, MASH with liver fibrosis, cirrhosis).^6^

Given the importance of early diagnosis and recent changes in the nomenclature of this disease, this study aims to estimate the level of awareness of the disease among the general public, people with diabetes, and primary care providers (PCPs).

## METHODS

The survey for this study consisted of two sections: sociodemographic questions and items assessing awareness of MASLD and fatty liver disease (Supplemental Materials and Methods, http://links.lww.com/HC9/B970).

We recruited 5408 participants from the four largest cities in the United States: New York City, NY; Los Angeles, CA; Chicago, IL; and Houston, TX, respectively,^7^ via river sampling, telephone recruitment, direct mail invitations, and opt-in options. Weighting resulted in samples of adults in the general population (n=4000, n=1000 per city), self-reporting a diabetes diagnosis (n=1000, n=250 per city), and PCPs (n=800, n=200 per city). Data were collected on 5–13 September 2024 through online panels provided by Consensus Strategies.

Descriptive statistics summarize sample demographic characteristics, including age, gender, education level, and race/ethnicity. Frequency distributions and percentages estimate awareness of MASLD and fatty liver disease. Chi-square tests evaluate differences between groups. The Emerson College (USA) institutional review board approved this study in August 2024 (protocol 25-003-F-E-8/21). Written, informed consent was obtained from each participant before enrollment.

## RESULTS

### Awareness of MASLD

In the general population sample (n=4000), 18.7% reported having heard of the term “MASLD” (Table 1). Among cities, residents of New York showed the highest awareness at 21.2%, followed by Chicago (20.2%), Los Angeles (17.0%), and Houston (16.2%). Among individuals with diabetes, 37.8% reported familiarity with MASLD (Supplemental Table S1, http://links.lww.com/HC9/B970). Among PCPs, 54.7% reported awareness (Supplemental Table S2, http://links.lww.com/HC9/B970). Overall, ethnicity was not associated with awareness: 18.6% and 18.7% of Black/African American and Hispanic/Latino respondents, respectively, were aware of MASLD (70.4% and 67.0% unaware, respectively), compared with 19.8% of White/Caucasian respondents. Among those with less than a high school education, 4.7% were aware, 79.1% were not, and 16.2% were unsure. By insurance status, 23.6% of those with private insurance were aware, compared with 17.7% on Medicare and 4.4% of those without health insurance. Those aged 60+ years reported 9.7% awareness, compared with 27.9% in the 30–39 age group (Table 1).

**TABLE 1:** Awareness of MASLD and fatty liver disease among the general population

	Heard of MASLD				Heard of fatty liver disease			
	Yes	No	Unsure	*p*	Yes	No	Unsure	*p*
Sample (n=4000)	18.7	68.6	12.7		78.1	17.2	4.7	
City				*0.312*				*0.424*
Chicago	20.2	68.7	11.1		80.1	15.4	4.5	
Houston	16.2	69.3	14.5		78.9	17.4	3.8	
Los Angeles	17.0	69.4	13.6		74.7	19.0	6.4	
New York City	21.2	67.1	11.7		78.6	17.1	4.3	
Gender				*0.000*				*0.000*
Man	20.1	68.3	11.5		76.6	19.2	4.2	
Woman	17.3	69.4	13.2		79.8	15.5	4.6	
Other/Prefer not to say	14.3	34.7	51.0		53.0	5.1	41.9	
Ethnicity				*0.426*				*0.000*
Hispanic or Latino of any race	18.7	67.0	14.4		77.0	18.1	5.0	
White or Caucasian	19.8	69.7	10.6		85.7	10.8	3.5	
Black or African American	18.6	70.4	11.0		69.1	26.1	4.8	
Asian	17.5	69.0	13.5		79.5	12.8	7.7	
Other or multiple races	13.4	67.6	19.0		73.0	24.3	2.8	
Education				*0.000*				*0.000*
Less than High School	4.7	79.1	16.2		62.5	34.0	3.5	
High School or General Educational Development (GED) Diploma	13.5	70.7	15.8		68.6	24.9	6.5	
Some College	9.1	77.6	13.4		78.4	15.5	6.2	
Associate or Vocational Degree	18.3	67.9	13.8		85.3	11.7	3.0	
Bachelor Degree	19.3	69.6	11.1		83.0	12.1	4.9	
Post-Graduate Degree (eg, Masters, Lawyer, Doctor)	45.7	46.9	7.4		88.5	9.4	2.1	
Age				*0.000*				*0.020*
18–29	20.7	67.4	12.0		70.0	23.9	6.0	
30–39	27.9	62.5	9.6		77.1	18.7	4.2	
40–49	21.9	62.7	15.4		81.0	14.5	4.5	
50–59	13.7	74.2	12.1		81.9	13.0	5.0	
60+	9.7	75.7	14.7		82.2	13.9	3.9	
Insurance status				*0.000*				*0.000*
Private health insurance (eg, employer-based, Affordable Care Act (ACA) State Marketplaces)	23.6	65.4	11.1		86.6	10.7	2.7	
Medicare	17.7	71.2	11.0		81.7	14.5	3.8	
Medicaid	19.3	66.8	14.0		71.5	21.7	6.8	
Other health insurance	14.2	68.5	17.3		72.5	22.7	4.8	
None	4.4	81.7	13.9		62.1	29.4	8.5	

### Awareness of fatty liver disease

Awareness of the term “fatty liver disease” was significantly higher across all groups as compared to awareness of MASLD. In the general population, 78.1% had heard of fatty liver disease, with the highest awareness in Chicago (80.1%), followed by New York City (78.6%), Houston (78.9%), and Los Angeles (74.7%) (Table 1). Among people with diabetes, 85.4% reported awareness of fatty liver disease, with New York City showing the highest awareness (89.6%), followed by Chicago (84.7%), Houston (84.3%), and Los Angeles (83.0%) (Supplemental Table S1, http://links.lww.com/HC9/B970). Among PCPs, 86.6% were aware of fatty liver disease, with awareness levels ranging from 89.4% in Chicago to 88.3% in New York City, 86.3% in Houston, and 82.6% in Los Angeles (Supplemental Table S2, http://links.lww.com/HC9/B970). Among those with less than a high school education, 78.5% were aware, compared with 88.0% of those with an Associate or Vocational Degree. By insurance status, 86.6% of those with private health insurance were aware, compared with 81.7% of those on Medicare and 75.7% of those without health insurance. Those aged 60+ years reported 82.2% awareness, compared with 77.1% in the 30–39 age group (Table 1).

## DISCUSSION

The study findings highlight the limited awareness of the 2023 nomenclature change from NAFLD to MASLD, with fewer than one-fifth of the general population, approximately one-third of adults with diabetes, and only about half of PCPs having heard of the new term MASLD.^1^ In sharp contrast, awareness of the umbrella term “fatty liver disease,” which now encompasses MASLD through to alcohol-associated liver disease and has been used for over 4 decades, but previously excluded alcohol-associated liver disease, was high. Over three-quarters of respondents in the general population were aware of the disease, and even more among those with diabetes and PCPs. The relatively low awareness of the term MASLD among PCPs is concerning, given their role in identifying at-risk individuals, managing early-stage MASLD, and referring to specialists when necessary.^8^

The contrast in terminology awareness reflects the challenges of transitioning to new nomenclature, especially when the older term has been embedded in public and professional discourse for decades, often minimized as “just fatty liver.” Effective dissemination of MASLD will require coordinated efforts among healthcare organizations, policymakers, and the media to ensure that patients, at-risk groups, and providers understand the implications of MASLD, its risk factors, and its management strategies. These efforts should emphasize that the nomenclature shift aims to provide greater clarity regarding the disease’s metabolic origins while reducing the stigma associated with both the terms “fatty” and “nonalcoholic,” thereby supporting a more nuanced and targeted approach to prevention and care. Strengthening MASLD-related training and resources for PCPs could enhance their capacity to address the disease effectively within primary care settings.

Study limitations may include recall bias from self-reported data, selection bias favoring health-conscious or diagnosed individuals, and variability in awareness levels in cities with differing healthcare and information access, warranting further research.

## Supplementary Material
