# Supplementary material for: Awareness of metabolic dysfunction–associated steatotic liver disease (MASLD) in 4 major cities in the United States
Source: Hepatol Commun. 2025 May 6;9(5):e0704. doi: 10.1097/HC9.0000000000000704 (PMC12055070; doi:10.1097/HC9.0000000000000704)
Supplement: Supplementary file 1 [file hc9-9-e0704-s001.pdf]

Supplemental Digital Content

Lazarus J.V. et al, "Awareness of Metabolic Dysfunction-Associated Steatotic Liver Disease (MASLD) in Four Major Cities in the United States"

SDC Materials and Methods. Study questionnaire.

1. Age: [Numeric entry]

2. Gender
    1. Man
    2. Woman
    3. Other
    4. Prefer not to say

3. Education level
    1. Less than High School
    2. High School or General Educational Development (GED) Diploma
    3. Some College
    4. Associate or Vocational Degree
    5. Bachelor Degree
    6. Post-Graduate Degree (e.g., Masters, Lawyer, Doctor)

4. Race/Ethnicity
    1. Hispanic or Latino of any race
    2. White or Caucasian
    3. Black or African American
    4. Asian
    5. Other or multiple races

5. Do you have health insurance?
    1. Yes, private health insurance (e.g., employer-based, Affordable Care Act (ACA) State Marketplaces)
    2. Yes, Medicare

3. Yes, Medicaid
4. Yes, other type of insurance
5. No

6. Do you see a primary healthcare provider at least one time per year?
    1. Yes
    2. No
    3. Unsure

7. Is your primary healthcare provider a…?
    1. Physician
    2. Physician assistant
    3. Nurse practitioner
    4. Other/Unsure

8. Which of the following do you believe can be associated with liver disease? Select all that apply.
    1. A high-calorie diet
    2. High-cholesterol foods
    3. Ultra-processed foods
    4. A sedentary lifestyle
    5. Alcohol use
    6. Supplements, vitamins, and herbal products
    7. None of the above

9. Do you believe that physical exercise can prevent some liver diseases?
    1. Yes
    2. No

3. Unsure

10. Do you believe screening for liver health is necessary as part of an annual health check-up?
    1. Yes
    2. No
    3. Unsure

11. Have you heard of a test called “FIB-4” that measures fibrosis (scarring) of the liver?
    1. Yes
    2. No
    3. Unsure

12. Non-invasive tests (NIT) are methods to check for liver disease without a biopsy, which requires obtaining a sample of the liver tissue. NITs are usually simpler, quicker, and safer than a biopsy. Have you heard of any of the following NITs? Select all that apply.
    1. Transient Elastography (e.g., Fibroscan®)
    2. Magnetic Resonance Imaging (MRI)
    3. Ultrasound or ultrasound elastography
    4. Blood tests (e.g., FIB-4, APRI, ALT/AST levels, ELF)
    5. None of the above
    6. Unsure

13. Have you ever had a blood test to assess your liver?
    1. Yes
    2. No
    3. Unsure

14. Have you ever heard of metabolic dysfunction-associated steatotic liver disease (MASLD)?
    1. Yes

2. No
3. Unsure

15. Have you heard of fatty liver?
    1. Yes
    2. No *[Skip to 17]*
    3. Unsure

16. From which sources have you heard about fatty liver? (Select all that apply)
    1. My doctor or healthcare provider
    2. Government health agencies like the Centers for Disease Control and Prevention (CDC)
    3. Family and friends
    4. TV
    5. Radio or Newspapers
    6. Social media
    7. Health/medical websites (e.g., WebMD)
    8. Other

17. Do you believe children can get fatty liver disease?
    1. Yes
    2. No
    3. Unsure

18. Did you know fatty liver disease can lead to more serious liver conditions such as cirrhosis and liver cancer?
    1. Yes
    2. No
    3. Unsure

19. How common do you think MASLD (also known as non-alcohol related liver disease and fatty liver disease) is among the adult population?

1. 1 in 100 adults (1%)
2. 1 in 50 adults (2%)
3. 1 in 10 adults (10%)
4. 1 in 5 adults (20%)
5. 1 in 4 adults (25%)
6. 1 in 3 adults (33%)
7. 1 in 2 adults (50%)
8. 3 in 4 adults (75%)

20. Do you know which type of diabetes you have?
    1. Type 1
    2. Type 1.5, or latent autoimmune diabetes in adults (LADA)
    3. Type 2
    4. Gestational
    5. Unsure

21. Are you currently receiving treatment for diabetes? (Select all that apply)
    1. Insulin
    2. Metformin
    3. GLP-1 receptor agonists (e.g., semaglutide (Ozempic®, Rybelsus®), liraglutide (Victoza®), dulaglutide (Trulicity®))
    4. Dual GIP/GLP receptor agonist (e.g., tirzepatide (Mounjaro®, Zepbound®))
    5. SGLT2 inhibitors (e.g., empagliflozin (Jardiance®), dapagliflozin (Farxiga®), canagliflozin (Invokana®), ertugliflozin (Steglatro®))
    6. Sulfonylurea (e.g., glimepiride, glyburide, glipizide)
    7. Pioglitazone (e.g., Actos®)
    8. DPP4 inhibitors (e.g., sitagliptin (Januvia®), linagliptin(Tradjenta®), saxagliptin (e.g., Onglyza®))
    9. Other (including diet-control)

22. What is your HbA1c level?

1. <5.7%
2. 5.7-6.4%
3. 6.5% to 8.0%
4. 8.1% or higher
5. Unsure

23. Has your healthcare provider ever discussed the increased risk of liver disease among people living with diabetes?
    1. Yes
    2. No
    3. Unsure

24. Which of the following risks are you aware of as contributors to MASLD? (Select all that apply)
    1. Diabetes
    2. Cardiovascular disease
    3. Overweight/obesity
    4. Several types of cancer, including gastrointestinal

25. Are you a…?
    1. Physician
    2. Physician assistant
    3. Nurse practitioner
    4. Other

26. How long have you been in practice? [Numeric entry]

27. In what kind of practice do you work?
    1. Solo private practice
    2. Hospital-based

3. Federally-qualified health center (FQHC)
4. Other community clinic
5. Other

28. Do you routinely assess your patients with the following characteristics for liver disease? (Select all that apply)
    1. Patients who are overweight or obese
    2. Patients who have diabetes
    3. Patients who smoke
    4. Patients who report low levels of physical activity
    5. Patients with metabolic risk factors not listed above

29. Do you discuss the risk of liver fibrosis with any of your patients with the following characteristics? (Select all that apply)
    1. Patients who are overweight or obese
    2. Patients who have diabetes
    3. Patients who smoke
    4. Patients who report low levels of physical activity
    5. Patients with metabolic risk factors not listed above

30. How often do you consider MASLD in patients presenting with elevated liver enzymes?
    1. Never or rarely
    2. Sometimes
    3. Often
    4. Always

31. What management strategies do you recommend for patients diagnosed with MASLD? (Select all that apply)
    1. Lifestyle modification (e.g., diet, exercise)
    2. Weight loss with anti-obesity medications or bariatric surgery
    3. Pharmacotherapy to treat metabolic comorbidities (e.g., insulin sensitizers, lipid-lowering agents)
    4. Monitoring and regular follow-ups

5. Referral to a specialist
6. Other

32. What criteria do you use to decide whether to refer a patient with suspected MASLD to a specialist? (Select all that apply)
    1. Persistent elevated liver enzymes
    2. Presence of comorbid conditions (e.g., diabetes, obesity)
    3. Advanced fibrosis/cirrhosis on imaging
    4. Patient's request
    5. Other

33. Do you use any patient-reported outcomes (PROs) in the management of liver health? (Select all that apply)
    1. Quality of life (QoL) or health-related quality of life (HRQoL) scales
    2. Symptom severity scales (e.g., fatigue, abdominal pain)
    3. Mental health assessments (e.g., depression, anxiety)
    4. Other
    5. None

Supplemental Digital Content

Lazarus J.V. et al, “Awareness of Metabolic Dysfunction-Associated Steatotic Liver Disease (MASLD) in Four Major Cities in the United States”

SDC Table 1. Awareness of MASLD and fatty liver disease among adults with diabetes

	Heard of MASLD				Heard of fatty liver disease			
	Yes	No	Unsure	p	Yes	No	Unsure	p
Sample (n=1000)	37.8	50.4	11.8		85.4	12.0	2.6	
City				*0.020*				*0.622*
Chicago	30.6	61.7	7.8		84.7	11.3	4.0	
Houston	30.2	54.6	15.3		84.3	13.5	2.3	
Los Angeles	45.7	39.4	14.8		83.0	15.0	2.0	
New York City	44.6	46.0	9.4		89.6	8.4	2.0	
Gender				*0.121*				*0.047*
Man	41.1	45.8	13.1		83.5	13.8	2.7	
Woman	33.9	55.7	10.5		87.8	9.8	2.4	
Other/Prefer not to say	92.3	0.0	7.7		23.1	69.2	7.7	
Ethnicity				*0.427*				*0.037*
Hispanic or Latino of any race	39.9	45.7	14.4		84.9	13.1	2.0	
White or Caucasian	40.6	48.2	11.3		93.9	4.6	1.6	
Black or African American	31.4	58.1	10.6		78.1	17.0	5.0	
Asian	38.3	55.7	6.0		82.9	15.5	1.7	
Other or multiple races	19.5	66.2	14.3		70.7	21.6	7.7	
Education				*0.000*				*0.688*

Less than High School	19.9	62.0	18.1		78.5	20.3	1.2	
High School or General Educational Development (GED) Diploma	21.1	57.3	21.5		85.0	11.7	3.3	
Some College	18.0	68.9	13.1		87.7	10.3	2.0	
Associate or Vocational Degree	30.1	63.5	6.4		88.0	10.3	1.8	
Bachelor Degree	32.3	56.6	11.1		82.4	15.3	2.3	
Post-Graduate Degree (e.g., Masters, Lawyer, Doctor)	73.9	20.3	5.8		87.8	8.8	3.5	
Age				*0.000*				*0.009*
18-29	59.1	37.5	3.4		80.1	19.8	0.0	
30-39	61.1	33.2	5.7		86.0	12.0	2.1	
40-49	47.0	45.7	7.3		89.0	9.5	1.6	
50-59	28.0	53.2	18.9		93.6	4.6	1.9	
60+	8.0	71.6	20.4		81.7	12.8	5.6	
Diabetes Type				*0.000*				*0.006*
Type 1	60.8	31.2	8.0		85.8	10.7	3.5	
Type 1.5 (LADA)	71.8	27.8	0.4		73.4	26.6	0.0	
Type 2	26.7	59.0	14.3		87.6	9.5	2.9	
Gestational	57.0	40.3	2.6		94.4	2.8	2.8	
Unsure	24.4	56.9	18.8		74.2	25.1	0.7	
Insurance Status				*0.002*				*0.139*

Private health insurance (e.g., employer-based, Affordable Care Act (ACA) State Marketplaces)	46.6	45.4	8.0		85.8	11.9	2.3	
Medicare	22.1	62.8	15.1		89.5	7.4	0.3	
Medicaid	38.1	47.2	14.7		87.3	11.8	0.9	
Other health insurance	36.1	51.1	12.9		78.3	12.6	9.1	
None	12.0	70.6	17.4		75.7	19.9	4.4	

Supplemental Digital Content

Lazarus J.V. et al, “Awareness of Metabolic Dysfunction-Associated Steatotic Liver Disease (MASLD) in Four Major Cities in the United States”

SDC Table 2. Awareness of MASLD and fatty liver disease among primary care providers (PCP)

	Heard of MASLD				Heard of fatty liver disease			
	Yes	No	Unsure	p	Yes	No	Unsure	p
Sample (n=800)	54.7	39.0	6.3		86.6	10.6	2.8	
City				*0.023*				*0.320*
Chicago	67.2	30.9	2.0		89.4	8.9	1.8	
Houston	41.1	52.7	6.2		86.3	10.2	3.5	
Los Angeles	58.7	36.8	4.6		82.6	16.8	0.7	
New York City	51.8	35.6	12.6		88.3	6.7	5.1	
Gender				*0.060*				*0.005*
Man	60.3	36.8	3.0		84.9	13.8	1.3	
Woman	50.6	40.9	8.4		88.8	7.9	3.3	
Other/Prefer not to say	38.9	28.7	32.4		46.8	20.8	32.4	
Ethnicity				*0.309*				*0.000*
Hispanic or Latino of any race	53.2	40.2	6.6		84.5	11.3	4.2	
White or Caucasian	62.8	31.6	5.6		93.3	4.1	2.5	
Black or African American	49.3	42.3	8.5		72.9	26.1	1.1	
Asian	50.1	48.7	1.3		95.5	4.1	0.4	
Other or multiple races	47.9	29.1	23.0		86.1	0.0	13.9	
Age				*0.235*				*0.001*

18-29	52.2	38.7	9.1		74.4	18.5	7.1	
30-39	60.3	37.7	2.1		88.6	10.8	0.6	
40-49	65.2	29.6	5.1		88.8	9.3	1.9	
50-59	47.9	48.3	3.8		96.7	2.0	1.3	
60+	42.1	46.0	11.9		96.1	3.5	0.4	
Professional Role				*0.000*				*0.035*
Physician	81.3	18.3	0.5		91.5	8.5	0.0	
Physician Assistant	69.6	28.3	2.1		86.6	12.5	1.0	
Nurse Practitioner	52.1	32.8	15.1		80.8	8.6	10.7	
Other	39.5	53.1	7.5		86.5	11.5	2.0	
Practice Type				*0.016*				*0.003*
Solo Private Practice	51.1	45.3	3.6		80.3	19.7	0.0	
Hospital-Based	64.8	32.1	3.1		88.8	9.7	1.5	
Federally Qualified Health Center (FQHC)	66.6	26.2	7.3		91.9	3.9	4.2	
Other Community Clinic	48.2	41.8	10.0		94.6	4.8	0.6	
Other	31.5	53.4	15.1		81.4	9.2	9.4	
Years in Practice				*0.195*				*0.001*
0-2	50.5	40.6	9.0		74.1	19.0	7.0	
3-6	62.5	34.8	2.7		90.7	8.8	0.5	

7-11	63.4	31.0	5.6		87.5	10.3	2.2	
12-19	58.8	38.0	3.3		96.1	3.9	0.0	
20+	38.4	51.9	9.6		98.9	0.9	1.9	
